# Risk of medication-induced lactic acidosis and hyperlactatemia: a pharmacovigilance study of the United States Food and Drug Administration’s Adverse Event Reporting System database

**DOI:** 10.3389/fphar.2025.1555955

**Published:** 2025-04-08

**Authors:** Houci Yang, Haibin Dai, Xveying Chen, Jiaqi Huang, Fangzhou Miao, Jiani Lv, Jiali Zhang

**Affiliations:** ^1^ Department of Pharmacy, Second Affiliated Hospital, Zhejiang University School of Medicine, Hangzhou, China; ^2^ Department Pharmacy, The First Affiliated Hospital of Zhejiang Chinese Medical University, Zhejiang Hospital of Traditional Chinese Medicine, Hangzhou, China

**Keywords:** lactic acidosis, hyperlactatemia, pharmacovigilance, FAERS, adverse reaction

## Abstract

**Objective:**

Lactic acidosis and hyperlactatemia (LAHL) are predictors of poor clinical outcomes in critically ill patients. This research aimed to specify medications reported in association with LAHL, thus providing valuable insights into medication safety.

**Methods:**

Spontaneous reports were excavated from the United States Food and Drug Administration’s Adverse Event Reporting System (FAERS) database from Q1 2004 to Q2 2024. Adverse reaction signals of medication-induced lactic acidosis and hyperlactatemia (MILAHL) were detected by reporting odds ratio (ROR) and proportional reporting ratio (PRR).

**Results:**

1,055 medications were identified as primary suspect medications of LAHL from Q1 2004 to Q2 2024, of which 180 were considered to have risk signals by ROR and 160 by PRR. Metformin (16,439 cases), linezolid (815 cases), amlodipine (646 cases), salbutamol (531 cases), and paracetamol (417 cases) were the top 5 medications with the most cases of LAHL. Among the top 50 medications with the strongest ROR and PRR signal, 16 were systemic antivirals, and 13 were antidiabetics (9 containing metformin). 23 of the top 50 medications with the strongest ROR and PRR signal did not indicate the risk of LAHL in the Summary of Product Characteristics (SmPC).

**Conclusion:**

This study listed high-risk medications by ROR and PRR analysis, especially those without an LAHL warning in SmPC, to help health professionals identify MILAHL in case of elevated lactate and enhance medication safety monitoring.

## 1 Introduction

Lactic acidosis and hyperlactatemia (LAHL) commonly occur in critically ill patients and serve as a predictor of poor clinical outcomes (Haas et al., 2016; Gunnerson et al., 2006; Khosravani et al., 2009; Nichol et al., 2010; Zhang and Xu, 2014). Hyperlactatemia refers to blood lactate levels over 2 mmol/L. Lactic acidosis usually has lactate levels above 4 mmol/L (Causes of lactic acidosis - UpToDate, 2024). Generally speaking, lactic acidosis is a worse stage of hyperlactatemia with arterial blood pH < 7.35 (Suetrong and Walley, 2016). LAHL is common in patients with severe sepsis and is associated with significant morbidity and mortality in the intensive care unit (ICU). The incidence of LAHL in ICU is 45% among neuro/trauma patients, 41% among medical patients, 40% among other surgical patients, and 36% among cardiac surgical patients (Khosravani et al., 2009). If there is no apparent lactate clearance within 12 h, the fatality rates for severe LAHL would be significantly increased (Haas et al., 2016), e.g., the fatality rates for patients with lactate levels over 10 mmol/L for more than 24 h is 96.8% (Gharipour et al., 2021). LAHL is an independent risk factor for death (Khosravani et al., 2009). Blood lactate levels can also serve as a therapeutic target. A quicker normalization of elevated lactate indicates a lower risk of death (Zanza et al., 2022), and awareness of the etiologies of LAHL is the base for diagnosis and treatment.

There are L-lactate and D-lactate in the human body, and L-lactate is the predominant stereoisomer (Talasniemi et al., 2008; Connor et al., 2008). LAHL has a variety of etiologies and can be grouped as type A (L-Lactate), type B (L-Lactate), and type D (D-Lactate) (Suetrong and Walley, 2016; Smith et al., 2019). Type A LAHL is associated with hypoxemia; type B is unrelated to hypoxemia, but two types of LAHL often co-exist (Post, 2000). Type B is related to different mechanisms and is further subdivided (Suetrong and Walley, 2016; Zanza et al., 2022) into B1 [underlying diseases related, e.g., Vitamin B1 deficiency (Omura et al., 2024; Baldo et al., 2023; Thota et al., 2021)], B2 [drugs or toxins related (Smith et al., 2019)] and B3 [inborn errors of metabolism-related, e.g., pyruvate carboxylase enzyme deficiency (Sharif et al., 2022) and pyruvate dehydrogenase complex deficiency (Zhou et al., 2024)]. Specific gastrointestinal bacterial species lead to the increased production of D-lactate and cause type D LAHL (Pohanka, 2020). It is typically associated with jejunoileal bypass surgery, small bowel resection, or short bowel syndrome (Pohanka, 2020; Adeva-Andany et al., 2014; Bianchetti et al., 2018).

Medication-induced lactic acidosis and hyperlactatemia (MILAHL) is type B2 LAHL, which is a diagnosis of exclusion and possibly underestimated (Mizock, 1987; Chen et al., 2015). Lactic acidosis cannot be ruled out if lactate is below 4 mmol/L or blood PH is over 7.35. Some patients have a standard anion gap, increased lactate, or PH over 7.35 due to the coexistence of acid-base disorder (Donnan and Segar, 2019). 86% of cases occurred when United States Food and Drug Administration (FDA)-labeled doses were employed. In 40.8% of MILAHL cases, culprit medications were continued without a change, and 16% of MILAHL patients died (Smith et al., 2019). Besides LAHL caused by metformin and nucleoside/nucleotide reverse transcriptase inhibitors (NRTIs), of which the pathophysiology and incidence of LAHL are thoroughly studied, little attention is paid to MILAHL.

United States Food and Drug Administration’s Adverse Event Reporting System (FAERS) database is a spontaneously maintained system for collecting post-marketing adverse events of medications and therapeutic biologics from various sources, including physicians, pharmacists, manufacturers, patients, and other healthcare providers (Sakaeda et al., 2013; Noguchi et al., 2021). A multitude of medication signal mining studies were conducted due to its large amount of data and free access (Alomar et al., 2020). In this research, we aimed to comprehensively identify potential medications that might cause LAHL by mining FAERS. We also aimed to facilitate early diagnosis of MILAHL in clinical practice to prevent the worst clinical outcomes and optimize medication therapy.

## 2 Materials and methods

### 2.1 Data sources

Data for this study was extracted from adverse event reports in the FAERS open source database, downloaded as quarterly data (https://fis.fda.gov/extensions/FPD-QDE-FAERS/FPD-QDE-FAERS.html) from Q1 2004 to Q2 2024 into SAS 9.4 for data cleaning and analysis. FAERS database contains the following eight types of files: demographic information (DEMO), drug information (DRUG), indications for use (INDI), start and end dates for reported drugs (THER), adverse events (REAC), patient outcomes (OUTC), report sources (RPSR), and invalid reports (DELETED).

### 2.2 Data processing

According to FDA guidelines for deduplicating reports, the CASEID, FDA_DT, and PRIMARYID fields were extracted from the DEMO table and sorted by order. When multiple reports had the same CASEID, the latest FDA_DT value was retained, ensuring it contained the most current and comprehensive information. Additionally, if there were reports with identical CASEID and FDA_DT values, the one with the highest PRIMARYID was kept to preserve complete data integrity. Since Q1 2019, each quarterly data package has included a list of deleted reports to remove corresponding post-deduplicated entries.

### 2.3 Standardization of drug names and adverse reactions

In the FAERS database, reported adverse events and indications for drug use were codified according to the Medical Dictionary for Regulatory Activities (MedDRA 27.0). The data on drug exposure reported in the corresponding dataset would be standardized by the World Health Organization Drug Dictionary (March 2024). In this study, 3 preferred terms (PTs) were taken from the SMQ name of lactic acidosis (SMQ code 20000033) to define LAHL. They were blood lactic acid increased (PT 1005635), hyperlactacidaemia (PT 10020660), and lactic acidosis (PT 10023676).

### 2.4 Data analysis

To detect potential adverse event signals, we employed ROR and PRR methods (Sakaeda et al., 2013; Ooba and Kubota, 2010; Evans et al., 2001). This method was based on a two-by-two contingency table (Supplementary Table S1) and aimed to identify potential adverse event signals by comparing the proportion of target events associated with target medication to the proportion of target events associated with all other medications. A risk signal was considered while ROR > 1, the lower limit of the corresponding 95% confidence interval (95% CI) > 1, and the number of reports (a) ≥ 3. For the PRR method, the signal generation criteria included PRR ≥ 2, variance (χ2) > 4, and a ≥ 3.

The selected signals needed to meet the criteria of ROR or PRR, indicating a potential association between the medication and the event (Supplementary Table S2). Count data was described using case numbers and proportions. All statistical analyses and visualizations were performed in SAS 9.4.

## 3 Results

### 3.1 Descriptive analysis

**FIGURE 1:**
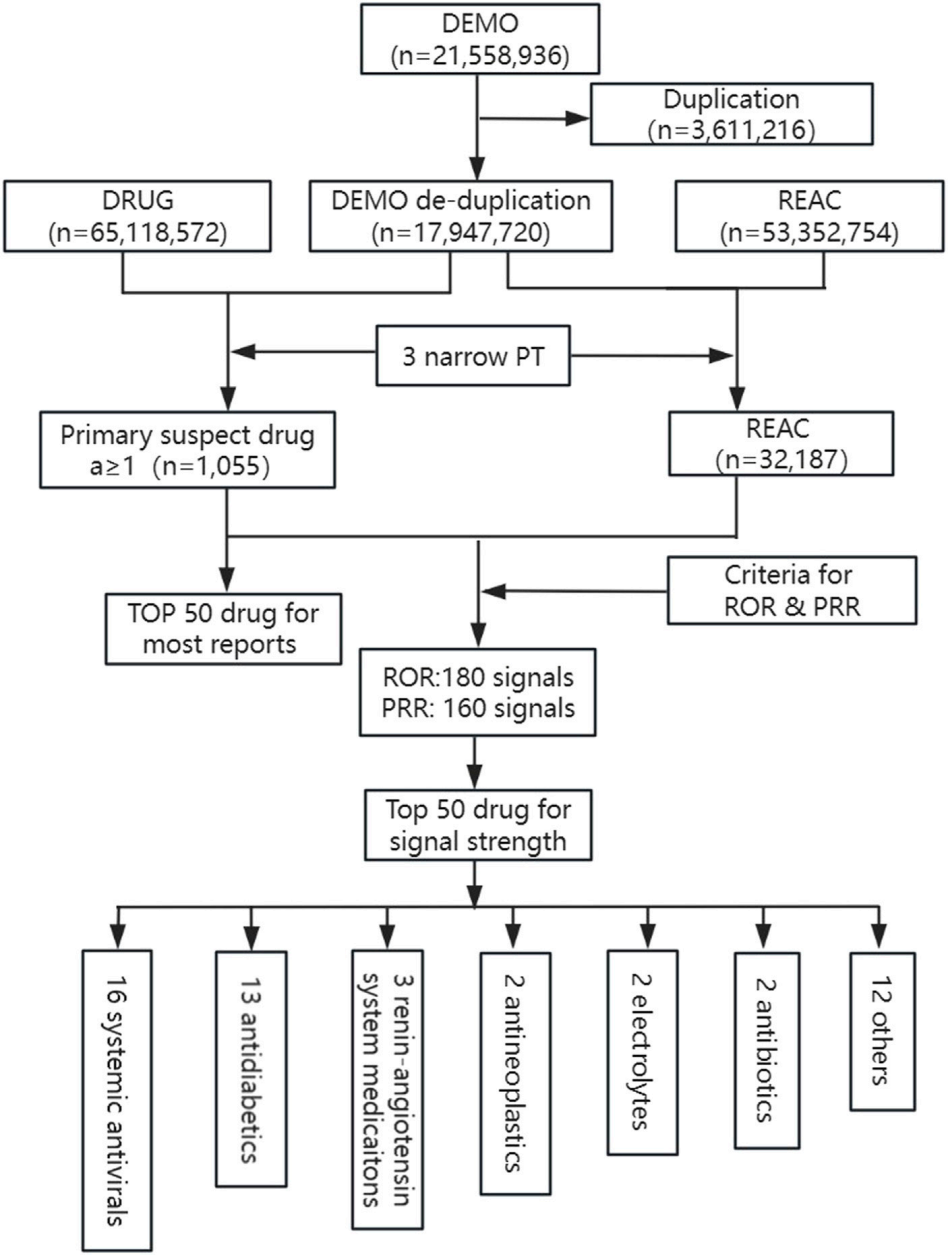
Flowchart of study process for identification of lactic acidosis and hyperlactatemia reports. DEMO, demographic information; DRUG, drug information; REAC, adverse events; PT, preferred terms; ROR, reporting odds ratio; PRR, proportional reporting ratio.

32,187 post-deduplicated LAHL cases were reported from Q1 2004 to Q2 2024 by investigating FAERS, a combination for 3 narrow PTs (PT 10005635, n = 4,310; PT 10020660, n = 2,103; PT 10023676, n = 26,364). 1,055 medications were detected as primary suspected medications. This study investigated the top 50 medications with the highest number of LAHL and the top 50 medications with the most substantial signal strength. Figure 1 provides the detailed study process.

**FIGURE 2:**
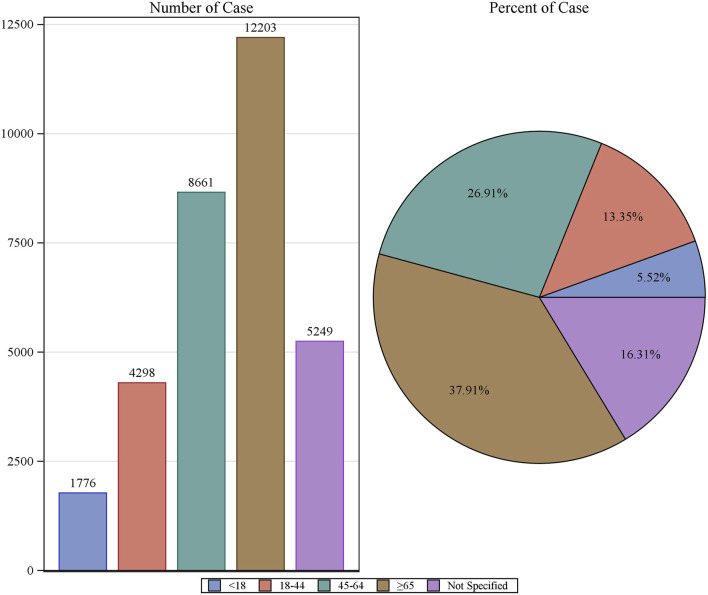
The number of reports on lactic acidosis and hyperlactatemia and the reporting rate by age.

**FIGURE 3:**
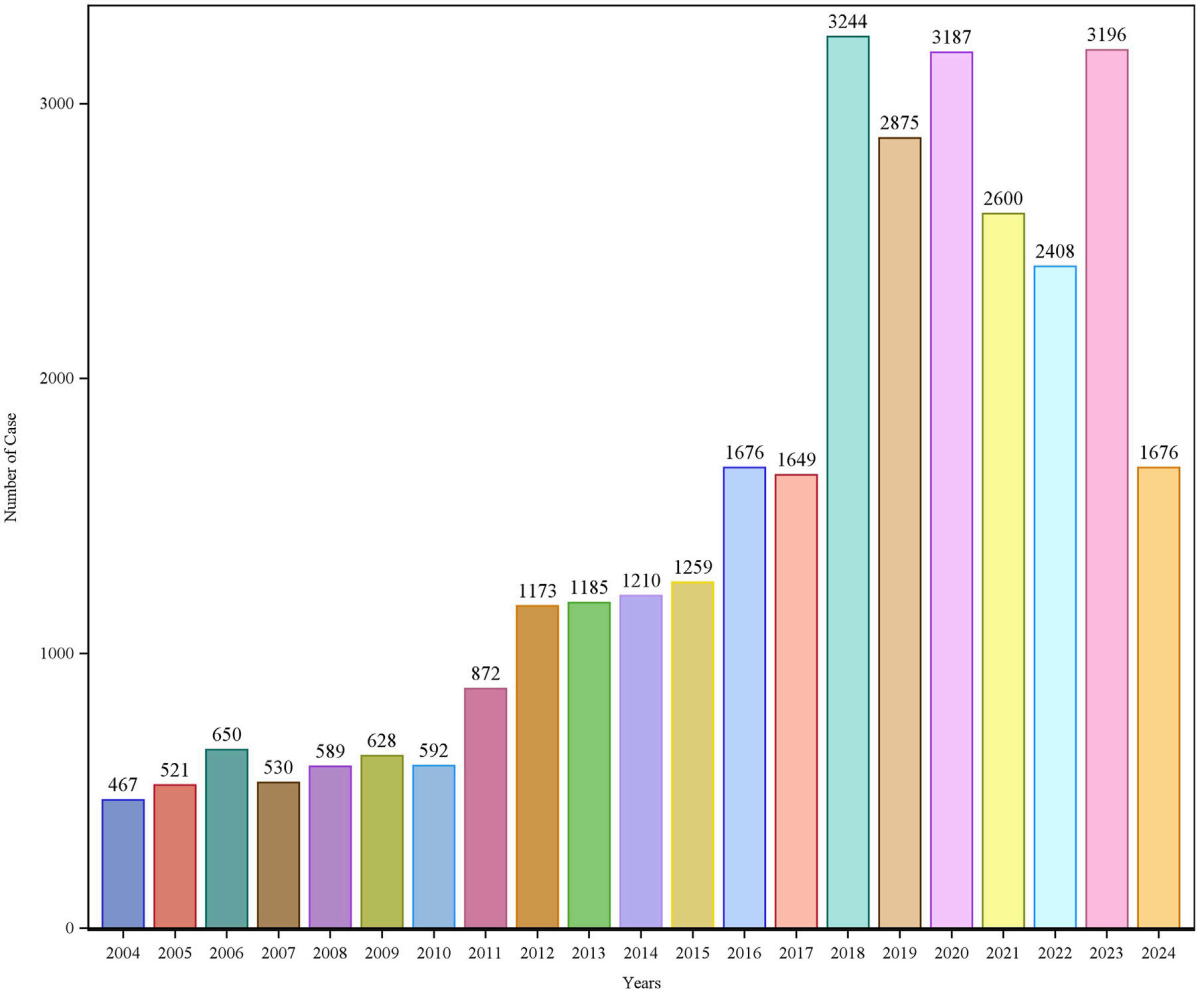
Lactic acidosis and hyperlactatemia reports by year.

**FIGURE 4:**
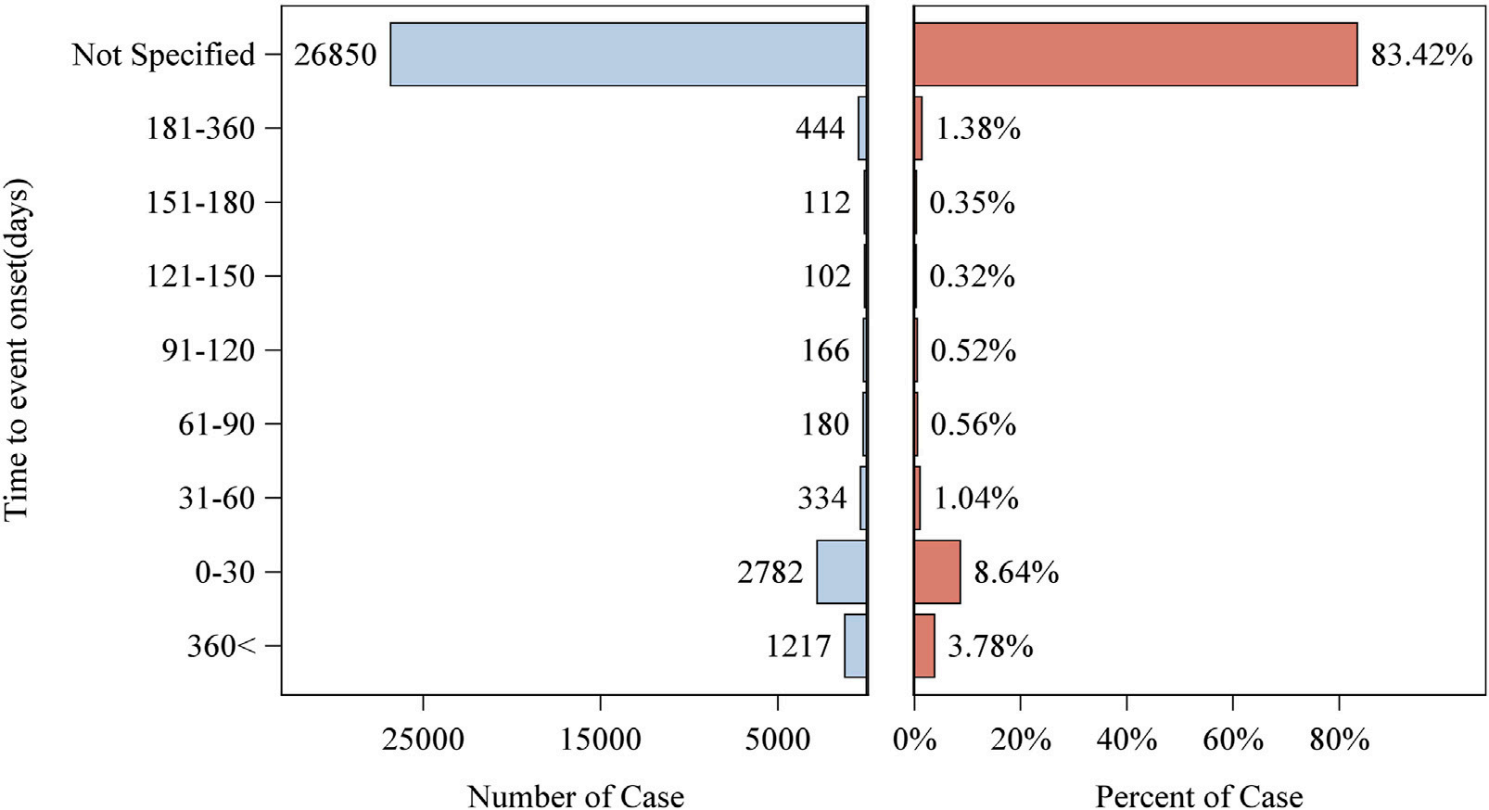
Reported lactic acidosis and hyperlactatemia onset time.

**FIGURE 5:**
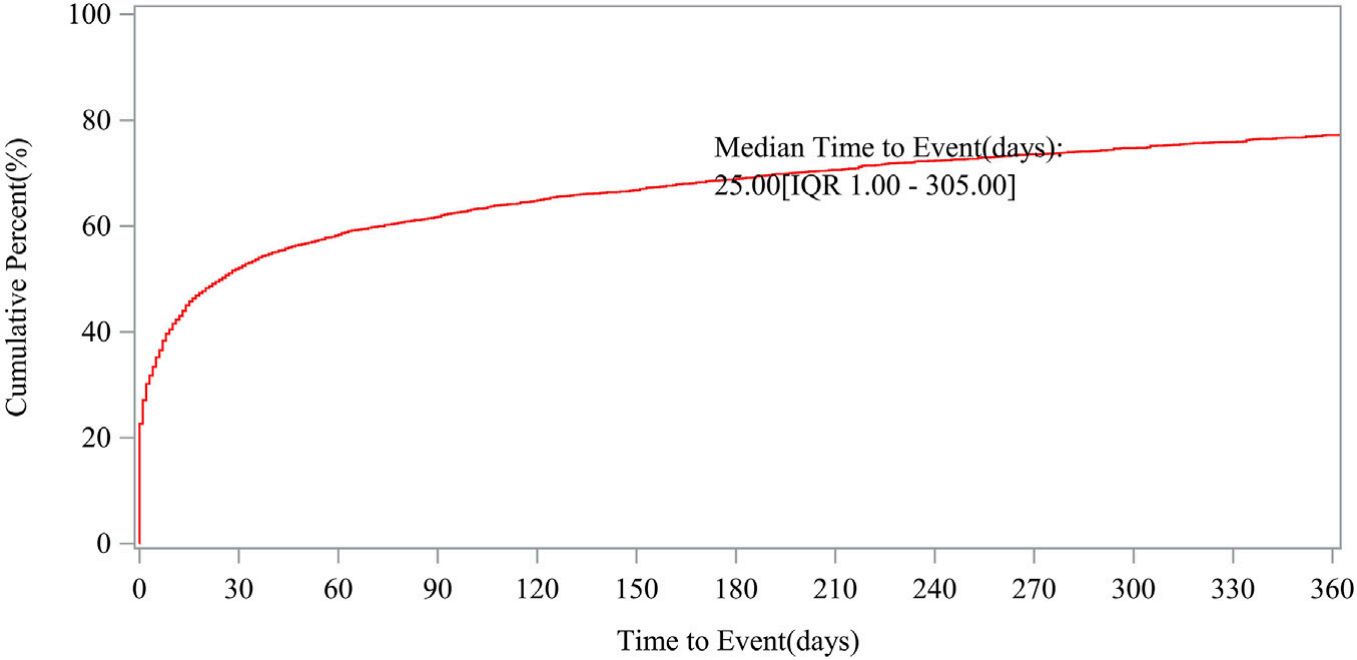
Median time to lactic acidosis and hyperlactatemia event.

Patient characteristics of LAHL are shown in Table 1; 32,187 cases were included. Besides those unknown in gender, the proportion of female reports (14,818 cases, 46.04%) was slightly higher than that of male reports (13,518 cases, 42.00%). The median age was 62 years old. As shown in Figure 2, the number of LAHL reports grew as age increased; patients over 65 (12,203 cases, 37.91%) had the highest risk of LAHL. Professionals reported the most cases compared with non-professionals: physicians reported 10,067 cases (31.28%), pharmacists 9,093 cases (28.25%), and other health professionals 8,506 cases (26.43%). The top 5 reporting countries were the United States of America, France, Germany, Italy, and the United Kingdom. Europe (16,717 cases, 51.94%) and North America (10,398 cases, 32.30%) were the primary reporting states. Most of the cases (31,957 cases, 99.29%) led to serious outcomes, including life-threatening (9,811 cases, 30.48%), initial or prolonged hospitalization (23,188 cases, 72.04%) and death (7,003 cases, 21.76%). The incidence rate of other outcomes is shown in Table 1. The number of LAHL reports had steadily grown, especially after the sharp increase in 2018 (Figure 3). Most of the reported onset time was within 30 days if it was specified (Figure 4), and the median time was 25 days (Figure 5).

**TABLE 1:** Patient characteristics from reports on medication-induced lactic acidosis and hyperlactatemia (n = 32,187).

Reporting information	Number (%)	Reporting information	Number (%)
Gender		Age	
Female	14,818 (46.04)	Median (Q1, Q3)	62.00 (47.00, 73.00)
Male	13,518 (42.00)	N (Missing)	26,938 (5,249)
Not specified	3,851 (11.96)	Country of the reporter	
Occupation of the reporter		United States of America	9,180 (28.52)
Consumer	2,745 (8.53)	France	5,374 (16.70)
Lawyer	46 (0.14)	Germany	2,475 (7.69)
Pharmacist	9,093 (28.25)	Italy	2,145 (6.66)
Physician	10,067 (31.28)	United Kingdom	1947 (6.05)
Other health-professional	8,506 (26.43)	Others	22,438 (30.29)
Not Specified	1730 (5.37)	Not Specified	1,317 (4.09)
Outcome		States of the reporter	
Life-Threatening	9,811 (30.48)	Europe	16,717 (51.94)
Hospitalization - Initial or Prolonged	23,188 (72.04)	North America	10,398 (32.30)
Disability	419 (1.30)	Asia	2,793 (8.68)
Death	7,003 (21.76)	Oceania	668 (2.08)
Congenital Anomaly	105 (0.33)	Africa	151 (0.47)
Required Intervention to Prevent Permanent Impairment/Damage	278 (0.86)	South America	143 (0.44)
Other serious medical events	17,157 (53.30)	Not Specified	1,317 (4.09)
Seriousness			
Serious	31,957 (99.29)		
Non-Serious	230 (0.71)		

### 3.2 LAHL medications

#### 3.2.1 Disproportionality analysis of the top 50 medications with the highest number of LAHL

**FIGURE 6:**
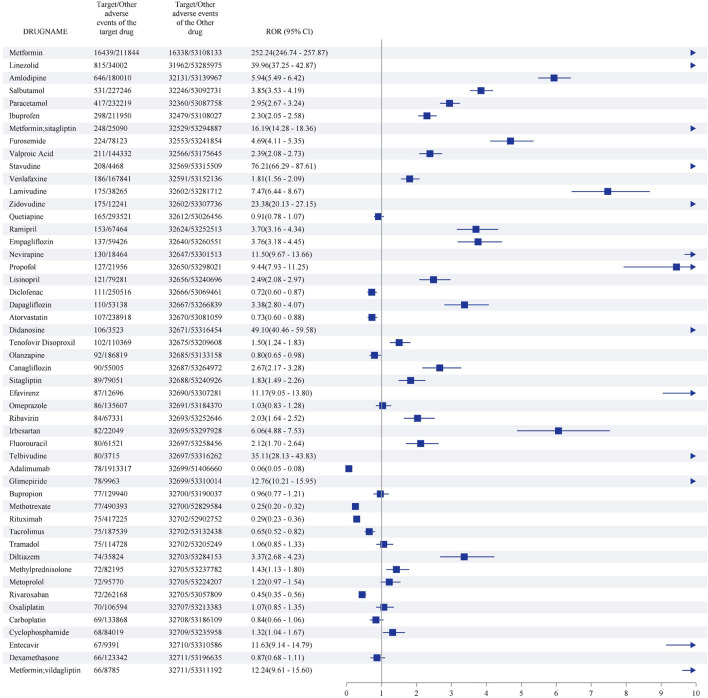
Top 50 medications with the highest number of lactic acidosis and hyperlactatemia.

The top 50 medications with the highest number of LAHL are shown in Figure 6. Metformin (16,439 cases) reported the most cases with the strongest signal (ROR 252.24, 95%CI 246.74–257.87), followed by linezolid (815 cases) with intense signal (ROR 39.96, 95%CI 37.25–42.87), amlodipine (646 cases) with positive signal (ROR 5.94, 95%CI 5.49–6.42), salbutamol (531 cases) with positive signal (ROR 3.85,95%CI 3.53–4.19), paracetamol (417 cases) with positive signal (ROR 2.95,95%CI 2.67–3.24). 35 out of the top 50 medications with the highest number of LAHL showed positive signals, including metformin/sitagliptin and metformin/vildagliptin, which were compound preparations with metformin.

#### 3.2.2 Analysis of the top 50 medications with the highest number of LAHL at the PT level

**FIGURE 7:**
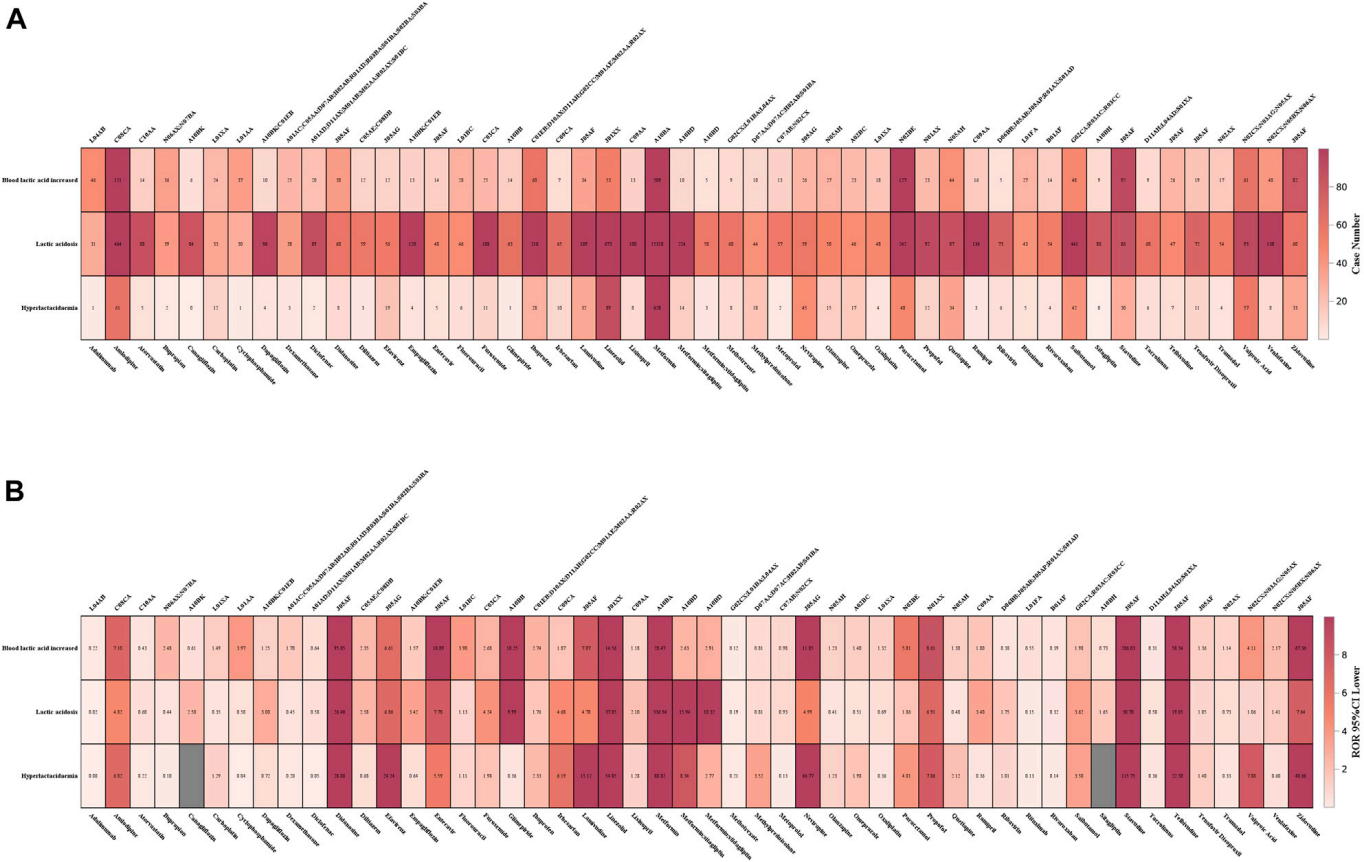
**(A)** Case numbers of the top 50 medications with the highest number of lactic acidosis and hyperlactatemia at the PT level. **(B)** 95%CI lower of the top 50 medications with the highest number of lactic acidosis and hyperlactatemia at the PT level. Tops words for **(A, B)** were ATC codification for the bottom medications.

The case number of the top 50 medications with the highest number of LAHL at the PT level was displayed in Figure 7A. Lactic acidosis (PT 10023676, n = 26,364) ranked top in three PTs, followed by blood lactic acid increased (BLAI, PT 10005635, n = 4,310) and hyperlactacidaemia (PT 10020660, n = 2,103). The middle part, lactic acidosis, was generally darker than BLAI in the upper part and hyperlactacidaemia in the lower part. However, the case number of BLAI of four medications (adalimumab, cyclophosphamide, stavudine, zidovudine) was more than that of lactic acidosis. For valproic acid, the combined case number of BLAI and hyperlactacidaemia was over that of lactic acidosis. The 5 medications mentioned above would increase blood lactate relatively mildly, leading to hyperlactatemia, while the other 45 medications were more likely to lead to lactic acidosis.

Figure 7B displayed the lower limit of the corresponding 95%CI of the top 50 medications with the highest number of LAHL at the PT level. Adalimumab, atorvastatin, diclofenac, methotrexate, metoprolol, rituximab, rivaroxaban, and tacrolimus were medications without any lower limit of 95%CI among three PTs over 1. Although these medications had many cases of LAHL, their ROR signals were negative.

### 3.3 Disproportionality analysis of the top 50 medications with the most substantial signal strength

1,055 primary suspect medications of LAHL were studied according to the criteria of ROR and PRR. 180 medications gave positive signals by ROR and 160 medications by PRR, as shown in Supplementary Tables S3, S4. ROR and PRR had their thresholds, and different signals were detected. However, 158 medications were detected with positive signals both by ROR and PRR. The higher the ROR or PRR value, the greater the LAHL risk. The top 50 medications with the most substantial ROR and PRR signal strength were consistent, as listed in Table 2. The medications were categorized according to the fourth level of the anatomical therapeutic chemical classification system (ATC). Among the top 50 medicines with the most substantial signal strength, 16 (lamivudine/stavudine, lamivudine/nevirapine/stavudine, stavudine, didanosine, amprenavir, telbivudine, indinavir, zidovudine, saquinavir, nelfinavir, emtricitabine, entecavir, nevirapine, efavirenz, abacavir, fosamprenavir) were systemic antivirals, 13 (metformin, gliclazide, alogliptin/metformin, repaglinide, linagliptin/metformin, metformin/pioglitazone, glimepiride, glibenclamide/metformin, metformin/sitagliptin, metformin/vildagliptin, glibenclamide, empagliflozin/metformin and dapagliflozin/metformin) were antidiabetics, among which 9 containing metformin. The remaining categories were 3 compound of preparations angiotensin-converting enzyme inhibitors (ACEI, enalapril/hydrochlorothiazide, amlodipine/perindopril, hydrochlorothiazide/irbesartan), 2 antineoplastics (tebentafusp, capivasertib), 2 electrolytes (electrolytes nos, sodium acetate), 2 antibiotics (linezolid, tedizolid), and 12 others (pentobarbital, nitroprusside, benzoic acid/sodium phenylacetate, glycine, vasopressin, terbutaline, carglumic acid, hydroxocobalamin, cimetidine, propofol, norepinephrine, colchicine). They are shown in Figure 1.

**TABLE 2:** Top 50 medications with strongest signal strength.

Drug	Report	ROR (95%CI)	PRR (χ2)	SmPC
metformin	16,439	252.24 (246.74–257.87)	234.15 (1903496.45)	Y
lamivudine/stavudine	4	144.62 (52.01–402.13)	132.89 (523.86)	Y
electrolytes nos	3	119.04 (36.86–384.44)	110.99 (327.18)	Y
lamivudine/nevirapine/stavudine	36	97.71 (69.79–136.80)	92.24 (3,247.35)	Y
sodium acetate	3	78.72 (24.71–250.79)	75.13 (219.56)	N
stavudine	208	76.21 (66.29–87.61)	72.86 (14,657.68)	Y
pentobarbital	10	55.92 (29.77–105.04)	54.09 (521.29)	Y
nitroprusside	9	52.12 (26.83–101.22)	50.53 (437.10)	Y
didanosine	106	49.10 (40.46–59.58)	47.70 (4,833.32)	Y
linezolid	815	39.96 (37.25–42.87)	39.05 (29,482.30)	Y
benzoic acid/sodium phenylacetate	5	39.88 (16.42–96.84)	38.95 (184.95)	N
amprenavir	3	36.70 (11.69–115.24)	35.91 (101.87)	Y
glycine	3	35.37 (11.27–111.02)	34.64 (98.05)	N
telbivudine	80	35.11 (28.13–43.83)	34.40 (2,589.19)	Y
vasopressin	9	27.32 (14.14–52.80)	26.89 (224.40)	N
alogliptin/metformin	9	27.07 (14.01–52.31)	26.64 (222.20)	Y
indinavir	26	25.23 (17.12–37.16)	24.86 (595.18)	N
zidovudine	175	23.38 (20.13–27.15)	23.06 (3,675.71)	Y
saquinavir	21	23.29 (15.13–35.83)	22.97 (441.29)	N
terbutaline	11	22.43 (12.37–40.67)	22.14 (222.10)	Y
linagliptin/metformin	19	22.33 (14.20–35.12)	22.04 (381.64)	Y
metformin/pioglitazone	17	21.79 (13.50–35.16)	21.51 (332.52)	Y
nelfinavir	24	17.28 (11.56–25.84)	17.11 (363.96)	N
glibenclamide/metformin	19	17.19 (10.94–27.02)	17.02 (286.52)	Y
gliclazide	3	16.43 (5.27–51.25)	16.28 (43.04)	N
metformin/sitagliptin	248	16.19 (14.28–18.36)	16.05 (3,474.46)	Y
hydroxocobalamin	6	15.18 (6.79–33.92)	15.05 (78.74)	N
tebentafusp	7	13.91 (6.61–29.27)	13.80 (83.12)	N
repaglinide	27	13.85 (9.48–20.24)	13.74 (319.00)	N
emtricitabine	16	13.85 (8.47–22.66)	13.74 (189.07)	Y
enalapril/hydrochlorothiazide	11	13.23 (7.31–23.95)	13.13 (123.31)	N
tedizolid	8	12.98 (6.47–26.03)	12.88 (87.72)	Y
glimepiride	78	12.76 (10.21–15.95)	12.67 (837.12)	N
metformin/vildagliptin	66	12.24 (9.61–15.60)	12.16 (675.06)	Y
glibenclamide	43	11.80 (8.74–15.93)	11.72 (421.28)	N
entecavir	67	11.63 (9.14–14.79)	11.55 (644.89)	Y
nevirapine	130	11.50 (9.67–13.66)	11.42 (1,232.06)	N
carglumic acid	11	11.36 (6.28–20.55)	11.29 (103.15)	N
efavirenz	87	11.17 (9.05–13.80)	11.11 (798.35)	N
empagliflozin/metformin	31	11.15 (7.83–15.88)	11.08 (284.19)	Y
amlodipine/perindopril	9	11.14 (5.79–21.47)	11.08 (82.52)	N
abacavir	45	10.65 (7.94–14.28)	10.58 (390.26)	Y
cimetidine	16	9.95 (6.08–16.26)	9.89 (127.91)	N
capivasertib	4	9.77 (3.66–26.11)	9.72 (31.30)	N
propofol	127	9.44 (7.93–11.25)	9.39 (949.42)	Y
norepinephrine	24	9.29 (6.22–13.87)	9.24 (176.32)	Y
fosamprenavir	10	8.41 (4.52–15.66)	8.37 (64.93)	N
colchicine	55	8.04 (6.17–10.48)	8.01 (336.95)	N
dapagliflozin/metformin	20	7.75 (4.99–12.03)	7.72 (116.96)	Y
hydrochlorothiazide/irbesartan	35	7.49 (5.38–10.45)	7.46 (195.84)	N

### 3.4 Risk warning analysis of the top 50 medications with the most substantial signal strength

The risk warning of LAHL in Summary of Product Characteristics (SmPC) for the top 50 medications with the most substantial signal strength was recorded in the last column in Table 2. 23 of the top 50 medications with the most substantial signal strength did not indicate the risk of LAHL in SmPC. Sodium acetate was the only medication in the top 10 that did not indicate LAHL risk. 6 (indinavir, saquinavir, nelfinavir, nevirapine, efavirenz, fosamprenavir) out of 16 systemic antivirals were without LAHL warning. As for the 13 antidiabetics, since the ingredient metformin carried a black box warning for the risk of lactic acidosis, 9 (metformin and 8 compound preparations with metformin) were warned. Still, the other 4 medications (gliclazide, repaglinide, glimepiride, glibenclamide) did not indicate the LAHL risk. There were no LAHL risk warnings in SmPC for 3 compound preparations of ACEI (enalapril/hydrochlorothiazide, amlodipine/perindopril and hydrochlorothiazide/irbesartan), 2 antineoplastics (tebentafusp, capivasertib), and 7 others (benzoic acid/sodium phenylacetate, glycine, vasopressin, hydroxocobalamin, carglumic acid, cimetidine, colchicine).

## 4 Discussion

This research was the first and the most extensive study to assess MILAHL in real-world data by analyzing FAERS. Patient characteristics of MILAHL, medications with the highest number of MLAHL cases, and medications with the strongest association with LAHL were reported. At the PT level, lactic acidosis (PT 10023676, n = 26,364) accounted for 81.9% of total reports (32,187 cases). The total case number was less than the combination of 3 PTs since some cases reported more than one PT, e.g., a case reported both BLAI (PT 10005635) and hyperlactacidaemia (PT 10020660). Smith et al. (2019) identified 59 risk medications of LAHL to aid clinicians in diagnosing MILAHL based on searching PubMed. They reviewed 286 MILAHL cases from 101 articles, of which 183 reported lactic acidosis, accounting for 64.0%. However, the rate was 81.9% when Smith et al. analyzed 105 patients from 86 publications (75 case reports and 11 case series), including individual patient data, and 86 out of 105 MILAHL cases were lactic acidosis. Lactic acidosis was common when lactate was elevated due to medications. In this study, the median onset time was 25 days, overall mortality was 21.76% (n = 7,003), and the median age was 62. In contrast, in Smith et al.’s analysis of 86 publications, the median onset time was 3 days, overall mortality was 16% (n = 46), and the median age was 47. The current study was based on a larger MILAHL population and provided updated data on patient characteristics for MILAHL. Medications with the most substantial signal strength should attract health professionals’ attention, especially those not indicating the risk of LAHL in SmPC.

16 systemic antivirals in the top 50 medications with the most substantial signal strength could be divided into 4 groups according to the fourth levels of the ATC codes: 5 protease inhibitors (PIs) (amprenavir, indinavir, saquinavir, nelfinavir, fosamprenavir), 7 NRTIs (stavudine, didanosine, telbivudine, zidovudine, emtricitabine, entecavir, abacavir), 2 non-nucleoside reverse transcriptase inhibitors (NNRTIs, nevirapine, efavirenz) and 2 compound preparations of NRTI (lamivudine/stavudine, lamivudine/nevirapine/stavudine). Amprenavir, 7 NRTIs, and 2 compound preparations of NRTI indicated the risk of LAHL in SmPC. NRTIs’ mechanism of LAHL was reported years ago in that they interfered with mitochondrial protein synthesis and then led to anaerobic metabolism (Arenas-Pinto et al., 2003). In Bonnet’s case-control study, low creatinine clearance and low nadir CD4^+^ T lymphocyte count before the inception of NRTI therapy were the main risk factors for lactic acidosis (Bonnet et al., 2003). It was reported that females were a risk factor for LAHL (Arenas-Pinto et al., 2003; Bonnet et al., 2003; Dragovic and Jevtovic, 2012; Fabian et al., 2008). PIs and NRTIs would contribute to lipodystrophy syndrome, particularly if associated with LAHL (Carr et al., 2000). The other 4 PIs and 2 NNRTIs without LAHL risk caution in SmPC were seldom found in the report on LAHL (Blohm et al., 2017), and their mechanism needed further study.

Sulfonylurea drugs (gliclazide, glimepiride, glibenclamide) and non-sulfonylurea drugs (repaglinide) were newly discovered antidiabetics with a high risk of LAHL by mining FAERS. Hepatoxicity and impaired hepatic function are adverse events that might cause poor clearance of lactate and lead to LAHL. Thiazolidinediones, DPP4 inhibitors, and SGLT2 inhibitors were also seldom relevant to LAHL. SGLT2 inhibitors had been warned of the risk of diabetic ketoacidosis, in which situation lactic acidosis was common (Donnan and Segar, 2019). Metformin was the medicine with the most reported LAHL cases and the highest risk by ROR and PRR in this study. The mortality rate for metformin-associated lactic acidosis (MALA) was as high as 50% (DeFronzo et al., 2016). By interfering with hepatic mitochondrial respiration or inhibiting the utilization of lactate toward gluconeogenesis, metformin caused lactate to accumulate, especially in patients with impaired renal and hepatic function and with the presence of increased lactate production, such as sepsis, chronic heart failure, reduced tissue perfusion, or anoxia (DeFronzo et al., 2016). Metformin could be given to patients with an estimated glomerular filtration rate (eGFR) of no less than 45 mL/min/1.73 m^2^. MALA risk would possibly be increased if the patients had an eGFR of less than 30 mL/min/1.73 m^2^, in which case the FDA did not allow metformin (Orloff et al., 2021). Though one study illustrated an incidence of 37% for contrast-induced nephropathy in the context of interventional cardiac angiography among patients with a baseline serum creatinine ≥ 1.8 mg/dL (Gruberg et al., 2000), there is no need to discontinue metformin before and following intravenous contrast exposure in patients with eGFR > 30 mL/min/1.73 m^2^. For patients with relatively preserved renal function, metformin is also safe during percutaneous coronary interventions (Sakellariou et al., 2023): it is unnecessary to stop metformin if eGFR is above 60 mL/min/1.73 m^2^; more evidence was needed to test the safety for patients with an eGFR of 30–60 mL/min/1.73 m^2^, though no case of lactic acidosis was detected (Kao et al., 2022). It was reported that higher plasma metformin levels correlated with higher lactate levels (Boucaud-Maitre et al., 2016), and continuous veno-venous hemofiltration was recommended for MALA (Bruijstens et al., 2008; Arroyo et al., 2010; Keller et al., 2011; Vecchio and Protti, 2011). The Comparative Outcomes Study of Metformin Intervention versus Conventional Approach Study (COSMIC) suggested that metformin was safe if contraindications and warnings were cautioned (Cryer et al., 2005).

Current Surviving Sepsis Campaign guidelines recommend administering balanced solutions within the first 3 h of resuscitation for sepsis-related hypotension or LAHL (Moschopoulos et al., 2023). Although acetate could potentially influence cardiovascular function (Chapp et al., 2024; Schrander-vd Meer et al., 1999; Kirkendol et al., 1978; Jacob et al., 1997), PlasmaLyte, the acetate ringer’s solution (AR), could correct volume and electrolyte deficit and address acidosis (Rizoli, 2011). There was little evidence of acetate-related toxicity in the context of volume loading compared with renal replacement (Morgan, 2013). Recently, it is reported that AR could cause a quicker decline in lactate levels without marked side effects for fluid resuscitation in children with septic shock (Li et al., 2024) and in infants with biliary atresia compared with lactate ringer’s solution (LR) (Liu X. et al., 2021). Compared with the vast use in clinical practice, the number of cases of LAHL with sodium acetate and electrolytes was only 3 for each. Since no detailed medication was referred to electrolytes nos, related medications were taken for LR. LR would bring higher blood lactate levels than normal saline (NS) in patients undergoing lower gynecological surgery (Scheingraber et al., 1999) and in patients undergoing renal transplantation (Hadimioglu et al., 2008). Compared with PlasmaLyte, LR was observed to increase patients’ lactate levels (Weinberg et al., 2015; Priyanka et al., 2023a; Shin et al., 2011). Though both had a similar acid-base status (Priyanka et al., 2023b), PlasmaLyte had a better metabolic profile than LR or NS (Hadimioglu et al., 2008) and could decrease the length of hospitalization as well as the complications rate (Weinberg et al., 2015). It would be inappropriate to apply LR to patients with impaired liver function (Kierdorf et al., 1999; Ergin et al., 2016). Still, Ellekjaer reported that with very low quality and quantity evidence, acetate versus lactate did not significantly affect patient-centered outcomes (Ellekjaer et al., 2022).

The occurrence of pentobarbital-induced LAHL (10 cases, ROR 55.92) was thought to be associated with propylene glycol (PG), a pharmaceutical excipient. Pentobarbital sodium contained 40% v/v of PG (Miller et al., 2008). This excipient was more frequently related to benzodiazepines, such as diazepam (Wilson et al., 2005) and lorazepam (Wilson et al., 2005; Neale et al., 2005; Arroliga et al., 2004; Arbour, 1999; Cawley, 2001). Diazepam had 40% v/v of PG and lorazepam had 80% v/v (Blohm et al., 2017). In this study, diazepam reported 35 cases, and lorazepam reported 32 cases, whereas neither had a positive signal. Other medications (Blohm et al., 2017) with PG also had LAHL reports in this data mining: trimethoprim/sulfamethoxazole (TMP/SMX, 40% v/v, 53 cases, ROR 2.13, 95%CI 1.62–2.78), phenobarbital (70% v/v, 6 cases, ROR 4.61, 95%CI 2.07–10.29), digoxin (40% v/v, 12 cases, ROR 0.21, 95%CI 0.12–0.37), hydralazine (40% v/v, 4 cases, ROR 0.60, 95%CI 0.22–1.60), phenytoin (40% v/v, 7 cases, ROR 0.21, 95%CI 0.10–0.44), etomidate (35% v/v, 1 case, ROR 2.13, 95%CI 0.30–15.16) and esmolol (25% v/v, 1 case, ROR 2.85, 95%CI 0.40–20.2695), only the first two had positive signal. To prevent PG toxicity, some researchers have recommended limiting daily intravenous PG intake to 2.9 g/h or 69 g/day and applying a 50% reduction in the maximum dose in patients with risk factors (Lim et al., 2014), such as liver disease, renal failure, pregnancy, concomitant medications, and children conservatively (Zar et al., 2007). A case of TMP/SMX-induced severe lactic acidosis involved relatively low amounts of PG (162 g within 3 days), far below the considered toxic levels, indicating an individualized metabolism, excretion, and toxicity of PG (Bulathsinghala et al., 2016). Osmol gap could work as a surrogate marker for serum PG level; previously, an osmol gap over 10 was associated with PG level and toxicity (Barnes et al., 2006). Apart from medications, Fireball Whiskey with PG also possibly led to severe lactic acidosis (Ahmed et al., 2021). About 45% of PG was excreted unchanged by kidneys, and the liver metabolized the remaining PG into lactate (Wilson et al., 2000; Ruddick, 1972). Hepatic metabolization of lactate can possibly be impaired by inadequate blood flow, less transport of lactate, or poor conversion into pyruvate (Neale et al., 2005).

Linezolid (LZD) and tedizolid were oxazolidinone antibiotics approved by the FDA in 2000 and 2014, respectively. LZD was the medication with 81 reports, the second largest amount of LAHL cases, and came 10th in signal strength, while tedizolid reported 8 cases and ranked 32nd. Their mechanism of LAHL might be related to mitochondrial toxicity. LZD disrupted mitochondrial protein synthesis by acting on human mitochondrial 16S RNA, structurally similar to bacterial 23 S rRNA (Narita et al., 2007; Palenzuela et al., 2005; De Vriese et al., 2006; Leach et al., 2007; Wilson et al., 2008; Ippolito et al., 2008). It was also reported that LZD-induced LAHL was related to diminished global oxygen consumption and extraction, and these variations reflected selective inhibition of mitochondrial protein synthesis/translation with secondary mononuclear imbalance (Protti et al., 2016). In a systematic review of 23 studies with 129 patients, 21.7% died in the setting of LZD-induced LAHL (Akter et al., 2024). In other studies, the mortality rate was 25%–50% (Gatti et al., 2021; Mao et al., 2018; Im et al., 2015). Certain conditions would increase the risk of LAHL with a standard dose of 600 mg twice daily, such as advanced age (Liu T. et al., 2021; Cattaneo et al., 2016), impaired renal function (Cattaneo et al., 2016; Morata et al., 2016; Velez and Janech, 2010; Mori et al., 2018), and prolonged therapy (Im et al., 2015; Boutoille et al., 2009; Sawyer et al., 2014). In contrast, some considered renal impairment had no concern with the incidence of lactic acidosis but could affect severity and mortality (Im et al., 2015). Hepatic dysfunction could also be considered a risk factor for LAHL since blood LZD level was found to be 4–6 times higher in hepatic dysfunctional patients compared with regular patients (Im et al., 2015). It was beneficial to use therapeutic drug monitoring (TDM) to achieve efficacy and safety (Liu et al., 2024; Santini et al., 2017), especially in pediatric patients receiving prolonged courses of linezolid or patients with underlying hepatic or renal dysfunction (Su et al., 2011). Therapeutic doses of tedizolid might be less likely to cause LAHL than LZD (Su et al., 2011), but further investigations were needed (Roger et al., 2018). As far as drug interactions were concerned, tedizolid was thought to have a lower risk of serotonin syndrome than linezolid (Fang et al., 2024).

LAHL was found in approximately 20% of patients with high doses of selective ß2-agonists (Liedtke et al., 2019) via the mechanism of increasing lactate synthesis in skeletal muscle (Levy et al., 2008; Qvisth et al., 2008) and respiratory muscles (Liedtke et al., 2019; Mountain et al., 1990) or through reduced elimination caused by liver hypoperfusion (Bohn, 2007). Terbutaline (11 cases, ROR 22.43, 95%CI 12.37–40.67), salbutamol (531 cases, ROR 3.84, 95%CI 3.53–4.19), levosalbutamol (4 cases, ROR 1.38, 95%CI 0.52–3.68), ipratropium/salbutamol (27 cases, ROR 1.53, 95%CI 1.05–2.24), budesonide/formoterol (11 cases, ROR 0.15, 95%CI 0.08–0.26) and fluticasone/salmeterol (10 cases, ROR 0.12, 95%CI 0.06–0.23) were ß2-agonists mined with cases over three in this study. Terbutaline had the highest ROR value among ß2-agonists. Salbutamol was the most frequently prescribed and reported ß2-agonist (Lewis et al., 2014; Rodrigo and Rodrigo, 2005; Zitek et al., 2016; Reyes-Mondragon et al., 2016; Saadia et al., 2015; Dodda and Spiro, 2012; Meegada et al., 2020; Miranda et al., 2024), and its lactate concentration was dose-dependent and correlated with salbutamol blood level (Liedtke et al., 2019). About 98% of inhaled ß2-agonist cases were caused by short-acting beta-agonists (SABA), consistent with Katsumata’s research that very few cases of lactic acidosis had been caused by long-acting beta-agonists (LABA) (Katsumata et al., 2024).

Propofol infusion syndrome (PRIS) was a rare but fatal complication when propofol was applied, and mitochondrial disorder was a risk factor, especially when combined with critical illness and status epilepticus (Shimizu et al., 2020). PRIS was first reported in children (Hatch, 1999) and then in adults administrated with doses over 5 mg/kg/h and with a duration longer than 48 h (Kam and Cardone, 2007). The occurrence of PRIS for seriously ill adults with propofol over 24 h was approximately 1%, even at low doses, and the mortality rate was 18% (Roberts et al., 2009). However, an analysis of FDA MedWatch reported a 30% mortality of PRIS from 1,139 suspected cases (Fong et al., 2008). Hemphill et al. (2019) reviewed 168 cases of PRIS from 108 publications and found that mortality in children was independently connected with fever and hepatomegaly and in adults with electrocardiogram changes, hypotension, high potassium, traumatic brain injury, and a mean propofol infusion rate over 5 mg/kg/h. Continuous renal replacement therapy was recommended for PRIS (Hemphill et al., 2019).

In this study, norepinephrine reported 24 cases with ROR 9.29 (6.22–13.87), ranking 46th for signal strength, and epinephrine reported 55 cases with ROR 3.21 (2.46–4.18), ranking 121th. Both medications were warned of the risk of lactate elevating in SmPC. Epinephrine-induced LAHL was probably related to carbohydrate metabolism but not cellular hypoxia (Levy et al., 2003). Vasoconstrictor effects mediated by the α1-adrenergic receptor (α1-R) predominated after epinephrine was given with an increased dose. Epinephrine exhibited main metabolic effects and increased glucose and lactate levels by increasing hepatic glucose output. It also stimulates the skeletal muscle to release lactate for oxidation or gluconeogenesis (Chasiotis et al., 1983). Wutrich found that after 4 h of epinephrine administration, the higher the lactate increased by epinephrine, the better the prognosis, indicating the existence of a metabolic reserve (Wutrich et al., 2010). However, the persistence of hyperlactatemia in shock patients maintained a poor prognosis. Norepinephrine had a minor effect on carbohydrate metabolism and acted mainly on the peripheral α1-R. One prospective randomized clinical trial reported that epinephrine and norepinephrine-dobutamine did not induce the same effects on lactate metabolism, though they demonstrated similar effects on systemic hemodynamics (Levy et al., 1997).

Cyanide toxicity was a rare complication of sodium nitroprusside. Sodium nitroprusside released cyanide (Smith and Kruszyna, 1974; Ivankovich et al., 1978), then metabolized by hepatic rhodanese using thiosulfate, producing kidney-excreted thiocyanate. When surplus cyanide binds to cytochrome C oxidase, it inhibits oxidative phosphorylation and leads to anaerobic metabolism, thus creating lactic acid. During continued sodium nitroprusside administration over 2 μg/kg/min, cyanide production was considered to surpass the endogenous metabolic capability, but it could be enhanced by simultaneous administration of thiosulfate (Udeh et al., 2015; Abraham, 2010). Lockwood reported that sodium nitroprusside could still be safely used beyond the dose of 2 μg/kg/min; monitoring lactic acid instead of cyanide levels was also a reasonable measure to ensure safety (Abraham, 2010).

Tebentafusp, capivasertib, enalapril/hydrochlorothiazide, amlodipine/perindopril, hydrochlorothiazide/irbesartan, benzoic acid/sodium phenylacetate, glycine, vasopressin, hydroxocobalamin, carglumic acid, cimetidine and colchicine were newly uncovered medications generating strong LAHL signal by ROR and PRR from FAERS, and there were no related risk warnings in SmPC. Thanks to the large sample size of FAERS, rare adverse events that are difficult to detect in traditional epidemiological studies can be identified (Li et al., 2023).

This pharmacovigilance study is a provision of reference for discovering potential LAHL medications, but some limitations must be considered. Firstly, many MILAHL cases were not reported on account of the spontaneous reporting property of the FAERS database. Secondly, incomplete reporting happened because some reporters skipped data such as gender or age; sometimes, even false reporting existed. Thirdly, most reporters came from Western countries like the United States and France. Thus, limitations might occur in the generalization of conclusions among Asian countries. Fourthly, dosage information and blood lactate concentrations were not collected so that only associations could be revealed. Nevertheless, the FAERS database still works as a significant tool for pharmacovigilance analysis.

## 5 Conclusion

Serum lactate concentration is a clinical marker for illness severity and risk of mortality, and the outcomes of LAHL are usually severe. This study listed high-risk medications by ROR and PRR analysis, especially those without an LAHL warning in SmPC, to help health professionals identify MILAHL in case of elevated lactate and enhance medication safety monitoring.

## Data Availability

The original contributions presented in the study are included in the article/Supplementary Material, further inquiries can be directed to the corresponding authors.
