# Supplementary material for: Risk of medication-induced lactic acidosis and hyperlactatemia: a pharmacovigilance study of the United States Food and Drug Administration’s Adverse Event Reporting System database
Source: Front Pharmacol. 2025 Apr 8;16:1555955. doi: 10.3389/fphar.2025.1555955 (PMC12011842; doi:10.3389/fphar.2025.1555955)
Supplement: Supplementary file 1 [file Supplementaryfile1.docx]

Supplementary Material

# Supplementary Table S1 Two-by-two contingency table for disproportionality analysis

| **Item** | **Target adverse events reported** | **Other adverse events reported** | **Total** |
| --- | --- | --- | --- |
| **Target drugs** | a | b | a + b |
| **Other drugs** | c | d | c + d |
| **Total** | a + c | b + d | a + b + c + d |

a, number of reports containing both the target drug and LAHL; b, number of reports containing other adverse reactions (AE) of the target drug; c, number of reports containing LAHL of other drugs; d, number of reports containing other drugs and other AE.

# Supplementary Table S2 Calculation formulas and Criteria of ROR and PRR

| **Method** | **Calculation formula** | **Criteria** |
| --- | --- | --- |
| **ROR** | $ROR=\frac{a / c}{b / d}$ | a ≥ 3  ROR > 1  95%CI (lower limit) > 1 |
|  | $SE(lnROR)=\sqrt{\frac{1}{a}+\frac{1}{b}+\frac{1}{c}+\frac{1}{d}}$ |  |
|  | $95\%CI= e^{\ln\left( ROR \right)\pm1.96se}$ |  |
|  |  |  |
| **PRR** | $PRR=\frac{a / (a+b)}{c / (c+d)}$ | a ≥ 3  PRR ≥ 2 |
|  | $\chi2 =\frac{{(ad-bc)}^{2}(a+b+c+d)}{( a+b)(a+c)(c+d)(b+d)}$ | $\chi2\geq4$ |

a, number of reports containing both the target drug and LAHL; b, number of reports containing other adverse reactions (AE) of the target drug; c, number of reports containing LAHL of other drugs; d, number of reports containing other drugs and other AE.

# Supplementary Table S3 180 medications with positive signals by ROR

| **Medications** | **Number of cases** | **ROR** | **95% CI** |
| --- | --- | --- | --- |
| metformin | 16439 | 252.24 | 246.74-257.87 |
| lamivudine/stavudine | 4 | 144.62 | 52.01-402.13 |
| electrolytes nos | 3 | 119.04 | 36.86-384.44 |
| lamivudine/nevirapine/stavudine | 36 | 97.71 | 69.79-136.8 |
| sodium acetate | 3 | 78.72 | 24.71-250.79 |
| stavudine | 208 | 76.21 | 66.29-87.61 |
| pentobarbital | 10 | 55.92 | 29.77-105.04 |
| nitroprusside | 9 | 52.12 | 26.83-101.22 |
| didanosine | 106 | 49.10 | 40.46-59.58 |
| linezolid | 815 | 39.96 | 37.25-42.87 |
| benzoic acid/sodium phenylacetate | 5 | 39.88 | 16.42-96.84 |
| amprenavir | 3 | 36.70 | 11.69-115.24 |
| glycine | 3 | 35.37 | 11.27-111.02 |
| telbivudine | 80 | 35.11 | 28.13-43.83 |
| vasopressin | 9 | 27.32 | 14.14-52.8 |
| alogliptin/metformin | 9 | 27.07 | 14.01-52.31 |
| indinavir | 26 | 25.23 | 17.12-37.16 |
| zidovudine | 175 | 23.38 | 20.13-27.15 |
| saquinavir | 21 | 23.29 | 15.13-35.83 |
| terbutaline | 11 | 22.43 | 12.37-40.67 |
| linagliptin/metformin | 19 | 22.33 | 14.2-35.12 |
| metformin/pioglitazone | 17 | 21.79 | 13.5-35.16 |
| nelfinavir | 24 | 17.28 | 11.56-25.84 |
| glibenclamide/metformin | 19 | 17.19 | 10.94-27.02 |
| gliclazide | 3 | 16.43 | 5.27-51.25 |
| metformin/sitagliptin | 248 | 16.19 | 14.28-18.36 |
| hydroxocobalamin | 6 | 15.18 | 6.79-33.92 |
| tebentafusp | 7 | 13.91 | 6.61-29.27 |
| repaglinide | 27 | 13.85 | 9.48-20.24 |
| emtricitabine | 16 | 13.85 | 8.47-22.66 |
| enalapril/hydrochlorothiazide | 11 | 13.23 | 7.31-23.95 |
| tedizolid | 8 | 12.98 | 6.47-26.03 |
| glimepiride | 78 | 12.76 | 10.21-15.95 |
| metformin/vildagliptin | 66 | 12.24 | 9.61-15.6 |
| glibenclamide | 43 | 11.80 | 8.74-15.93 |
| entecavir | 67 | 11.63 | 9.14-14.79 |
| nevirapine | 130 | 11.50 | 9.67-13.66 |
| carglumic acid | 11 | 11.36 | 6.28-20.55 |
| efavirenz | 87 | 11.17 | 9.05-13.8 |
| empagliflozin/metformin | 31 | 11.15 | 7.83-15.88 |
| amlodipine/perindopril | 9 | 11.14 | 5.79-21.47 |
| abacavir | 45 | 10.65 | 7.94-14.28 |
| cimetidine | 16 | 9.95 | 6.08-16.26 |
| capivasertib | 4 | 9.77 | 3.66-26.11 |
| propofol | 127 | 9.44 | 7.93-11.25 |
| norepinephrine | 24 | 9.29 | 6.22-13.87 |
| fosamprenavir | 10 | 8.41 | 4.52-15.66 |
| colchicine | 55 | 8.04 | 6.17-10.48 |
| dapagliflozin/metformin | 20 | 7.75 | 4.99-12.03 |
| hydrochlorothiazide/irbesartan | 35 | 7.49 | 5.38-10.45 |
| lamivudine | 175 | 7.47 | 6.44-8.67 |
| glucose/heparin | 4 | 7.41 | 2.78-19.79 |
| atracurium | 8 | 7.29 | 3.64-14.6 |
| metformin/saxagliptin | 15 | 7.21 | 4.34-11.97 |
| acarbose | 15 | 6.60 | 3.97-10.96 |
| factor i (fibrinogen) | 3 | 6.37 | 2.05-19.8 |
| canagliflozin/metformin | 9 | 6.12 | 3.18-11.78 |
| irbesartan | 82 | 6.06 | 4.88-7.53 |
| amlodipine | 646 | 5.94 | 5.49-6.42 |
| perindopril | 41 | 5.93 | 4.36-8.06 |
| methylthioninium | 4 | 5.73 | 2.15-15.29 |
| ketoprofen | 13 | 5.72 | 3.32-9.86 |
| sodium bicarbonate | 6 | 5.66 | 2.54-12.62 |
| tenofovir alafenamide | 14 | 5.52 | 3.26-9.32 |
| sodium phenylbutyrate | 3 | 5.51 | 1.77-17.11 |
| lamivudine/zidovudine | 37 | 5.50 | 3.98-7.6 |
| trandolapril/verapamil | 6 | 5.45 | 2.45-12.16 |
| amlodipine/olmesartan | 9 | 5.45 | 2.83-10.48 |
| isoflurane | 5 | 5.36 | 2.23-12.89 |
| betaxolol | 3 | 5.21 | 1.68-16.18 |
| calaspargase pegol | 3 | 5.06 | 1.63-15.71 |
| tenofovir | 13 | 4.85 | 2.81-8.35 |
| sevoflurane | 27 | 4.84 | 3.31-7.06 |
| hydrochlorothiazide/telmisartan | 13 | 4.82 | 2.8-8.31 |
| acetylcysteine | 7 | 4.82 | 2.29-10.11 |
| ritonavir | 62 | 4.81 | 3.75-6.18 |
| trandolapril | 6 | 4.76 | 2.14-10.62 |
| anidulafungin | 3 | 4.72 | 1.52-14.67 |
| furosemide | 224 | 4.69 | 4.11-5.35 |
| phenobarbital | 6 | 4.62 | 2.07-10.29 |
| verapamil | 66 | 4.57 | 3.59-5.82 |
| abacavir/lamivudine/zidovudine | 7 | 4.57 | 2.17-9.59 |
| isoniazid | 22 | 4.55 | 2.99-6.92 |
| cobicistat/darunavir | 5 | 4.43 | 1.84-10.66 |
| rasburicase | 4 | 4.42 | 1.66-11.8 |
| lopinavir/ritonavir | 59 | 4.42 | 3.42-5.7 |
| theophylline | 19 | 4.39 | 2.8-6.89 |
| tagraxofusp | 3 | 4.32 | 1.39-13.41 |
| abacavir/lamivudine | 38 | 4.09 | 2.97-5.62 |
| bictegravir/emtricitabine/tenofovir alafenamide | 29 | 4.07 | 2.83-5.86 |
| cefotaxime | 9 | 4.06 | 2.11-7.81 |
| enfuvirtide | 10 | 4.04 | 2.17-7.52 |
| etravirine | 6 | 4.00 | 1.79-8.91 |
| spironolactone | 64 | 3.92 | 3.06-5.01 |
| desflurane | 3 | 3.90 | 1.26-12.11 |
| tranexamic acid | 12 | 3.88 | 2.2-6.83 |
| magnesium sulfate | 7 | 3.87 | 1.84-8.13 |
| salbutamol | 531 | 3.85 | 3.53-4.19 |
| empagliflozin | 137 | 3.76 | 3.18-4.45 |
| ramipril | 153 | 3.70 | 3.16-4.34 |
| hydrochlorothiazide/triamterene | 9 | 3.68 | 1.91-7.08 |
| prazosin | 4 | 3.66 | 1.37-9.77 |
| enalapril | 40 | 3.66 | 2.69-5 |
| fosinopril | 3 | 3.62 | 1.17-11.23 |
| bleomycin | 9 | 3.54 | 1.84-6.81 |
| atazanavir | 23 | 3.53 | 2.34-5.31 |
| cobicistat/elvitegravir/emtricitabine/tenofovir alafenamide | 11 | 3.53 | 1.95-6.37 |
| daunorubicin | 13 | 3.49 | 2.02-6.01 |
| glucagon | 5 | 3.49 | 1.45-8.39 |
| diphenhydramine/paracetamol | 9 | 3.48 | 1.81-6.69 |
| candesartan/hydrochlorothiazide | 13 | 3.47 | 2.01-5.98 |
| metformin/rosiglitazone | 20 | 3.47 | 2.24-5.38 |
| hydrochlorothiazide | 60 | 3.45 | 2.68-4.45 |
| mercaptopurine | 19 | 3.41 | 2.17-5.34 |
| sulfadiazine | 3 | 3.38 | 1.09-10.49 |
| dapagliflozin | 110 | 3.38 | 2.8-4.07 |
| diltiazem | 74 | 3.37 | 2.68-4.23 |
| emtricitabine/tenofovir alafenamide | 7 | 3.32 | 1.58-6.96 |
| pentoxifylline | 4 | 3.29 | 1.23-8.77 |
| dalbavancin | 4 | 3.23 | 1.21-8.61 |
| epinephrine | 55 | 3.21 | 2.46-4.18 |
| nifedipine | 31 | 3.18 | 2.23-4.52 |
| hydrochlorothiazide/lisinopril | 15 | 3.17 | 1.91-5.26 |
| nebivolol | 23 | 3.17 | 2.11-4.78 |
| atropine | 7 | 3.02 | 1.44-6.34 |
| midodrine | 4 | 2.98 | 1.12-7.96 |
| paracetamol | 417 | 2.95 | 2.67-3.24 |
| ganciclovir | 11 | 2.88 | 1.59-5.2 |
| ceftriaxone | 52 | 2.79 | 2.12-3.66 |
| iodixanol | 11 | 2.77 | 1.53-5 |
| daratumumab/vorhyaluronidase alfa | 5 | 2.72 | 1.13-6.54 |
| pegaspargase | 22 | 2.72 | 1.79-4.13 |
| idecabtagene vicleucel | 4 | 2.71 | 1.02-7.22 |
| glipizide | 15 | 2.70 | 1.63-4.48 |
| hydrochlorothiazide/losartan | 16 | 2.69 | 1.65-4.4 |
| dolutegravir | 23 | 2.67 | 1.78-4.02 |
| canagliflozin | 90 | 2.67 | 2.17-3.28 |
| elotuzumab | 6 | 2.66 | 1.19-5.93 |
| tisagenlecleucel | 24 | 2.65 | 1.77-3.95 |
| atenolol | 57 | 2.60 | 2.01-3.38 |
| ipratropium | 21 | 2.51 | 1.64-3.85 |
| lisinopril | 121 | 2.49 | 2.08-2.97 |
| dapsone | 6 | 2.39 | 1.07-5.33 |
| valproic acid | 211 | 2.39 | 2.08-2.73 |
| ibuprofen | 298 | 2.30 | 2.05-2.58 |
| allopurinol | 44 | 2.29 | 1.71-3.08 |
| cytarabine | 64 | 2.22 | 1.74-2.84 |
| meropenem | 16 | 2.21 | 1.35-3.6 |
| adefovir | 11 | 2.19 | 1.21-3.97 |
| torasemide | 12 | 2.15 | 1.22-3.79 |
| sulfamethoxazole/trimethoprim | 53 | 2.13 | 1.62-2.78 |
| fluorouracil | 80 | 2.12 | 1.7-2.64 |
| ivabradine | 9 | 2.12 | 1.1-4.07 |
| indapamide | 9 | 2.06 | 1.07-3.97 |
| candesartan | 36 | 2.06 | 1.49-2.86 |
| darunavir | 12 | 2.05 | 1.16-3.61 |
| ribavirin | 84 | 2.03 | 1.64-2.52 |
| brentuximab vedotin | 26 | 2.01 | 1.37-2.96 |
| telmisartan | 21 | 1.99 | 1.29-3.05 |
| bisoprolol | 52 | 1.98 | 1.5-2.59 |
| pravastatin | 23 | 1.96 | 1.3-2.95 |
| raltegravir | 16 | 1.96 | 1.2-3.2 |
| amitriptyline | 22 | 1.96 | 1.29-2.98 |
| saxagliptin | 9 | 1.95 | 1.02-3.76 |
| propafenone | 11 | 1.93 | 1.07-3.49 |
| daptomycin | 24 | 1.91 | 1.28-2.85 |
| sitagliptin | 89 | 1.83 | 1.49-2.26 |
| venlafaxine | 186 | 1.81 | 1.56-2.09 |
| antithymocyte immunoglobulin | 26 | 1.77 | 1.2-2.6 |
| remdesivir | 19 | 1.75 | 1.12-2.75 |
| rifampicin | 24 | 1.71 | 1.14-2.55 |
| linagliptin | 17 | 1.66 | 1.03-2.67 |
| teduglutide | 43 | 1.65 | 1.22-2.22 |
| ipratropium/salbutamol | 27 | 1.54 | 1.05-2.24 |
| tenofovir disoproxil | 102 | 1.50 | 1.24-1.83 |
| methylprednisolone | 72 | 1.43 | 1.13-1.8 |
| azithromycin | 63 | 1.39 | 1.08-1.78 |
| mirtazapine | 56 | 1.36 | 1.05-1.77 |
| amiodarone | 63 | 1.34 | 1.05-1.72 |
| cyclophosphamide | 68 | 1.32 | 1.04-1.67 |

# Supplementary Table S4 160 medications with positive signals by RRR

| **Medications** | **Number of reports** | **PRR value** | **Chi-Square**  **(χ2)** |
| --- | --- | --- | --- |
| metformin | 16439 | 234.15 | 1903496.45 |
| lamivudine/stavudine | 4 | 132.89 | 523.86 |
| electrolytes nos | 3 | 110.99 | 327.18 |
| lamivudine/nevirapine/stavudine | 36 | 92.24 | 3247.35 |
| sodium acetate | 3 | 75.13 | 219.56 |
| stavudine | 208 | 72.86 | 14657.68 |
| pentobarbital | 10 | 54.09 | 521.29 |
| nitroprusside | 9 | 50.53 | 437.1 |
| didanosine | 106 | 47.7 | 4833.32 |
| linezolid | 815 | 39.05 | 29482.3 |
| benzoic acid/sodium phenylacetate | 5 | 38.95 | 184.95 |
| amprenavir | 3 | 35.91 | 101.87 |
| glycine | 3 | 34.64 | 98.05 |
| telbivudine | 80 | 34.4 | 2589.19 |
| vasopressin | 9 | 26.89 | 224.4 |
| alogliptin/metformin | 9 | 26.64 | 222.2 |
| indinavir | 26 | 24.86 | 595.18 |
| zidovudine | 175 | 23.06 | 3675.71 |
| saquinavir | 21 | 22.97 | 441.29 |
| terbutaline | 11 | 22.14 | 222.1 |
| linagliptin/metformin | 19 | 22.04 | 381.64 |
| metformin/pioglitazone | 17 | 21.51 | 332.52 |
| nelfinavir | 24 | 17.11 | 363.96 |
| glibenclamide/metformin | 19 | 17.02 | 286.52 |
| gliclazide | 3 | 16.28 | 43.04 |
| metformin/sitagliptin | 248 | 16.05 | 3474.46 |
| hydroxocobalamin | 6 | 15.05 | 78.74 |
| tebentafusp | 7 | 13.8 | 83.12 |
| repaglinide | 27 | 13.74 | 319 |
| emtricitabine | 16 | 13.74 | 189.07 |
| enalapril/hydrochlorothiazide | 11 | 13.13 | 123.31 |
| tedizolid | 8 | 12.88 | 87.72 |
| glimepiride | 78 | 12.67 | 837.12 |
| metformin/vildagliptin | 66 | 12.16 | 675.06 |
| glibenclamide | 43 | 11.72 | 421.28 |
| entecavir | 67 | 11.55 | 644.89 |
| nevirapine | 130 | 11.42 | 1232.06 |
| carglumic acid | 11 | 11.29 | 103.15 |
| efavirenz | 87 | 11.11 | 798.35 |
| empagliflozin/metformin | 31 | 11.08 | 284.19 |
| amlodipine/perindopril | 9 | 11.08 | 82.52 |
| abacavir | 45 | 10.58 | 390.26 |
| cimetidine | 16 | 9.89 | 127.91 |
| capivasertib | 4 | 9.72 | 31.3 |
| propofol | 127 | 9.39 | 949.42 |
| norepinephrine | 24 | 9.24 | 176.32 |
| fosamprenavir | 10 | 8.37 | 64.93 |
| colchicine | 55 | 8.01 | 336.95 |
| dapagliflozin/metformin | 20 | 7.72 | 116.96 |
| hydrochlorothiazide/irbesartan | 35 | 7.46 | 195.84 |
| lamivudine | 175 | 7.44 | 971.73 |
| glucose/heparin | 4 | 7.38 | 22.08 |
| atracurium | 8 | 7.26 | 43.2 |
| metformin/saxagliptin | 15 | 7.18 | 79.83 |
| acarbose | 15 | 6.58 | 70.94 |
| factor i (fibrinogen) | 3 | 6.35 | 13.53 |
| canagliflozin/metformin | 9 | 6.1 | 38.39 |
| irbesartan | 82 | 6.04 | 344.51 |
| amlodipine | 646 | 5.92 | 2589.45 |
| perindopril | 41 | 5.91 | 167.25 |
| methylthioninium | 4 | 5.71 | 15.56 |
| ketoprofen | 13 | 5.7 | 50.42 |
| sodium bicarbonate | 6 | 5.65 | 22.95 |
| tenofovir alafenamide | 14 | 5.5 | 51.57 |
| sodium phenylbutyrate | 3 | 5.49 | 11.03 |
| lamivudine/zidovudine | 37 | 5.49 | 135.75 |
| trandolapril/verapamil | 6 | 5.44 | 21.74 |
| amlodipine/olmesartan | 9 | 5.43 | 32.55 |
| isoflurane | 5 | 5.34 | 17.67 |
| betaxolol | 3 | 5.2 | 10.17 |
| calaspargase pegol | 3 | 5.05 | 9.73 |
| tenofovir | 13 | 4.83 | 39.54 |
| sevoflurane | 27 | 4.82 | 81.83 |
| hydrochlorothiazide/telmisartan | 13 | 4.81 | 39.24 |
| acetylcysteine | 7 | 4.8 | 21.1 |
| ritonavir | 62 | 4.8 | 186.33 |
| trandolapril | 6 | 4.75 | 17.79 |
| anidulafungin | 3 | 4.71 | 8.78 |
| furosemide | 224 | 4.68 | 643.94 |
| phenobarbital | 6 | 4.61 | 16.94 |
| verapamil | 66 | 4.56 | 183.18 |
| abacavir/lamivudine/zidovudine | 7 | 4.56 | 19.44 |
| isoniazid | 22 | 4.54 | 60.75 |
| cobicistat/darunavir | 5 | 4.42 | 13.26 |
| rasburicase | 4 | 4.41 | 10.57 |
| lopinavir/ritonavir | 59 | 4.41 | 155.23 |
| theophylline | 19 | 4.38 | 49.55 |
| tagraxofusp | 3 | 4.31 | 7.63 |
| abacavir/lamivudine | 38 | 4.08 | 88.31 |
| bictegravir/emtricitabine/tenofovir alafenamide | 29 | 4.06 | 66.93 |
| cefotaxime | 9 | 4.05 | 20.69 |
| enfuvirtide | 10 | 4.03 | 22.83 |
| etravirine | 6 | 3.99 | 13.45 |
| spironolactone | 64 | 3.91 | 138.45 |
| desflurane | 3 | 3.89 | 6.46 |
| tranexamic acid | 12 | 3.87 | 25.53 |
| magnesium sulfate | 7 | 3.87 | 14.88 |
| salbutamol | 531 | 3.84 | 1098.26 |
| empagliflozin | 137 | 3.76 | 275.99 |
| ramipril | 153 | 3.7 | 299.63 |
| hydrochlorothiazide/triamterene | 9 | 3.68 | 17.53 |
| prazosin | 4 | 3.66 | 7.73 |
| enalapril | 40 | 3.66 | 77.19 |
| fosinopril | 3 | 3.61 | 5.67 |
| bleomycin | 9 | 3.53 | 16.36 |
| atazanavir | 23 | 3.52 | 41.53 |
| cobicistat/elvitegravir/emtricitabine/tenofovir alafenamide | 11 | 3.52 | 19.86 |
| daunorubicin | 13 | 3.48 | 23.02 |
| glucagon | 5 | 3.48 | 8.85 |
| diphenhydramine/paracetamol | 9 | 3.47 | 15.85 |
| candesartan/hydrochlorothiazide | 13 | 3.47 | 22.81 |
| metformin/rosiglitazone | 20 | 3.46 | 35.07 |
| hydrochlorothiazide | 60 | 3.45 | 104.13 |
| mercaptopurine | 19 | 3.4 | 32.22 |
| sulfadiazine | 3 | 3.37 | 5.01 |
| dapagliflozin | 110 | 3.37 | 182.89 |
| diltiazem | 74 | 3.36 | 122.51 |
| emtricitabine/tenofovir alafenamide | 7 | 3.31 | 11.29 |
| pentoxifylline | 4 | 3.28 | 6.36 |
| dalbavancin | 4 | 3.22 | 6.14 |
| epinephrine | 55 | 3.21 | 83.38 |
| nifedipine | 31 | 3.17 | 46.14 |
| hydrochlorothiazide/lisinopril | 15 | 3.17 | 22.26 |
| nebivolol | 23 | 3.17 | 34.11 |
| caffeine | 3 | 3.04 | 4.11 |
| ethosuximide | 3 | 3.02 | 4.06 |
| atropine | 7 | 3.02 | 9.44 |
| midodrine | 4 | 2.98 | 5.27 |
| paracetamol | 417 | 2.94 | 528.24 |
| ganciclovir | 11 | 2.88 | 13.46 |
| ceftriaxone | 52 | 2.78 | 59.41 |
| iodixanol | 11 | 2.77 | 12.41 |
| daratumumab/vorhyaluronidase alfa | 5 | 2.71 | 5.42 |
| pegaspargase | 22 | 2.71 | 23.79 |
| idecabtagene vicleucel | 4 | 2.7 | 4.3 |
| glipizide | 15 | 2.7 | 16 |
| hydrochlorothiazide/losartan | 16 | 2.69 | 17 |
| dolutegravir | 23 | 2.67 | 24.03 |
| canagliflozin | 90 | 2.66 | 93.31 |
| elotuzumab | 6 | 2.66 | 6.21 |
| tisagenlecleucel | 24 | 2.64 | 24.55 |
| atenolol | 57 | 2.6 | 56.09 |
| ipratropium | 21 | 2.51 | 19.02 |
| lisinopril | 121 | 2.49 | 107.15 |
| dapsone | 6 | 2.39 | 4.85 |
| valproic acid | 211 | 2.39 | 168.73 |
| ibuprofen | 298 | 2.3 | 216.43 |
| allopurinol | 44 | 2.29 | 32 |
| cytarabine | 64 | 2.22 | 42.79 |
| meropenem | 16 | 2.2 | 10.53 |
| adefovir | 11 | 2.19 | 7.14 |
| torasemide | 12 | 2.15 | 7.38 |
| sulfamethoxazole/trimethoprim | 53 | 2.12 | 31.52 |
| fluorouracil | 80 | 2.12 | 47.04 |
| ivabradine | 9 | 2.12 | 5.3 |
| indapamide | 9 | 2.06 | 4.92 |
| candesartan | 36 | 2.06 | 19.58 |
| darunavir | 12 | 2.05 | 6.45 |
| ribavirin | 84 | 2.03 | 43.87 |
| brentuximab vedotin | 26 | 2.01 | 13.21 |
